# Data on cryptogamic biota in relation to heavy metal concentrations in soil

**DOI:** 10.1016/j.dib.2018.05.137

**Published:** 2018-05-31

**Authors:** Kaja Rola, Piotr Osyczka

**Affiliations:** Institute of Botany, Faculty of Biology, Jagiellonian University, Gronostajowa 3, 30-387 Kraków, Poland

**Keywords:** Lichens, Bryophytes, Cryptogamic biota structure, Zn–Pb ores, Post-industrial areas, Environmental assessment

## Abstract

The data presented here are related to the research article entitled “Cryptogamic communities as a useful bioindication tool for estimating the degree of soil pollution with heavy metals” (Rola and Osyczka, 2018) [1]. These data concern the relationships between epigeic cryptogamic biota and heavy metal concentrations in soil of areas associated with Zn–Pb industry. The presence of particular species and coverage of lichens and bryophytes as well as soil chemical parameters in relation to three different soil pollution classes and five habitat types are provided. Included data could be used to compare cryptogamic community structure and pollutant concentration levels with other Zn–Pb polluted areas.

**Specifications Table**

**Table:** 

Subject area	*Environmental pollution*
More specific subject area	*Soil pollution, Cryptogamic biota*
Type of data	*Table, figure*
How data was acquired	*The presence and coverage of lichen and bryophyte species were determined in study plots.*
	*The following soil parameters were analysed: pH (electrometrically determined, Hach Lange HQ40d pH meter), organic carbon content (dry combustion technique, LECO SC-144DR Analyzer), total nitrogen content (the Kjeldahl method, Kjeltec 2300 Analyzer Unit), concentrations of total and exchangeable forms of Zn, Pb, Cd and As (FAAS, Varian Fast Sequential Atomic Absorption Spectrometer 280 and Varian Zeeman Atomic Absorption Spectrometer 280 with Graphite Tube Atomizer 120).*
Data format	*Raw, processed*
Experimental factors	*Soil samples designated for chemical analyses were dried and passed through a 2-mm sieve. For measurements of total metal element concentrations samples were digested with 70% HClO*_*4*_*. Extracting with a 0.05-M EDTA solution was applied for exchangeable forms of elements determination.*
Experimental features	*210 plots of 1 m×1 m were analysed in terms of cryptogamic biota. From 72 plots corresponding soil samples were collected for chemical analyses.*
Data source location	*Various types of anthropogenic and semi-natural sites directly associated with the processing of Zn–Pb ores in southern Poland*
Data accessibility	*Data are included in this article*
Related research article	*K. Rola, P. Osyczka, Cryptogamic communities as a useful bioindication tool for estimating the degree of soil pollution with heavy metals, Ecol. Indic. 88 (2018) 454–464.*

**Value of the data**

• Provided data may serve as a benchmark for bioindication studies based on the characteristics of cryptogamic biota.
• This data could be used to compare cryptogamic community structure in other Zn–Pb polluted areas.
• Data shown here can be useful for the planning of restoration projects, reclamation interventions, or conservation strategies.
• Data can be used as a base-line data for metal concentration levels in soils within areas associated with Zn–Pb industry.

## 1 Data

**Fig. 1:**
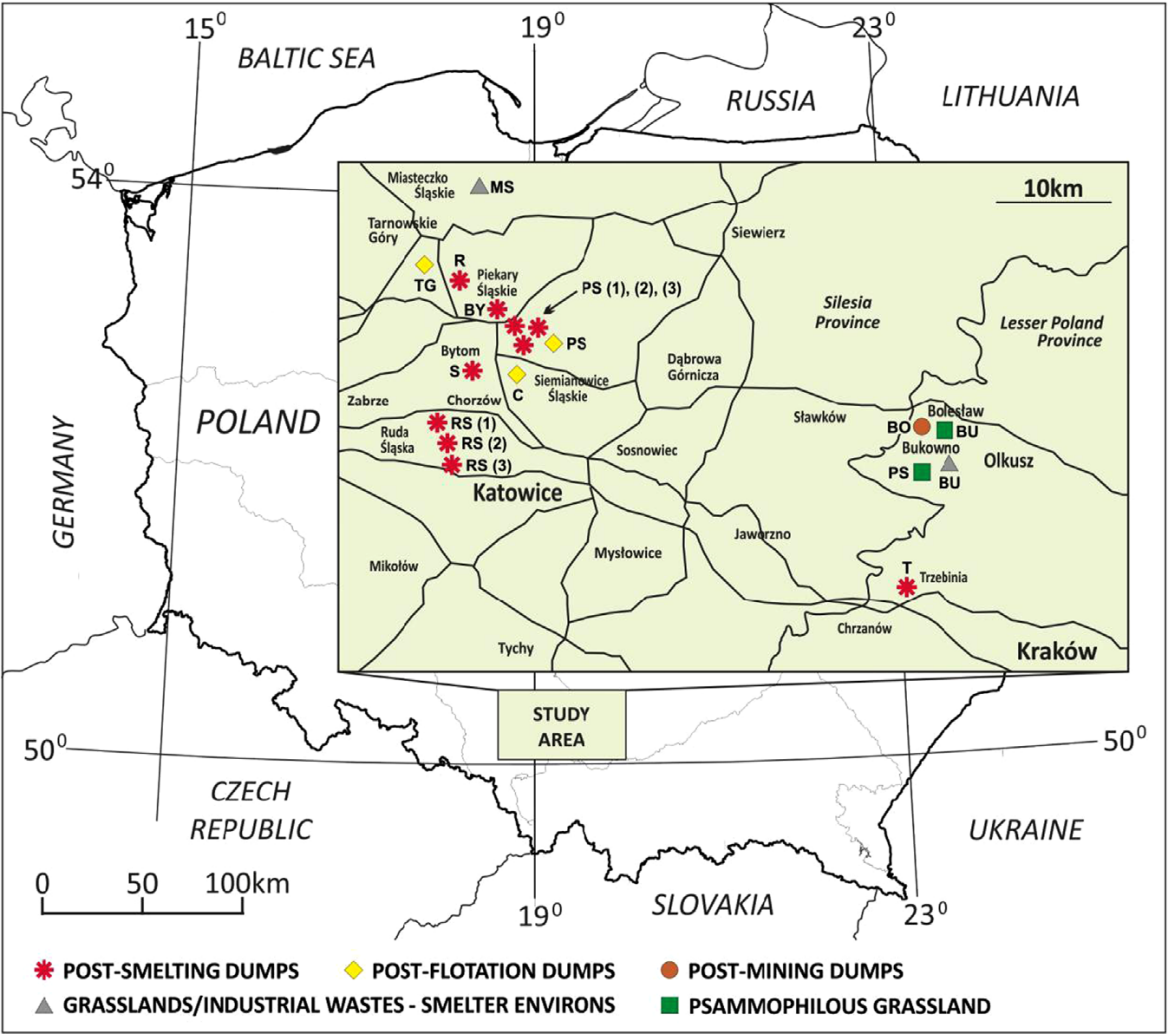
Location of the study sites in the Silesia-Cracow Upland (Poland). The type of habitat for particular sites are provided on the map. Abbreviations of study sites are as follow: BO – Bolesław, BU – Bukowno, BY – Bytom, C – Chorzów, MS – Miasteczko Śląskie, PS – Piekary Śląskie, PS (green square) – Pustynia Starczynowska, R – Radzionków, RS – Ruda Śląska, S – Świętochłowice, T – Trzebinia, TG – Tarnowskie Góry.

**Fig. 2:**
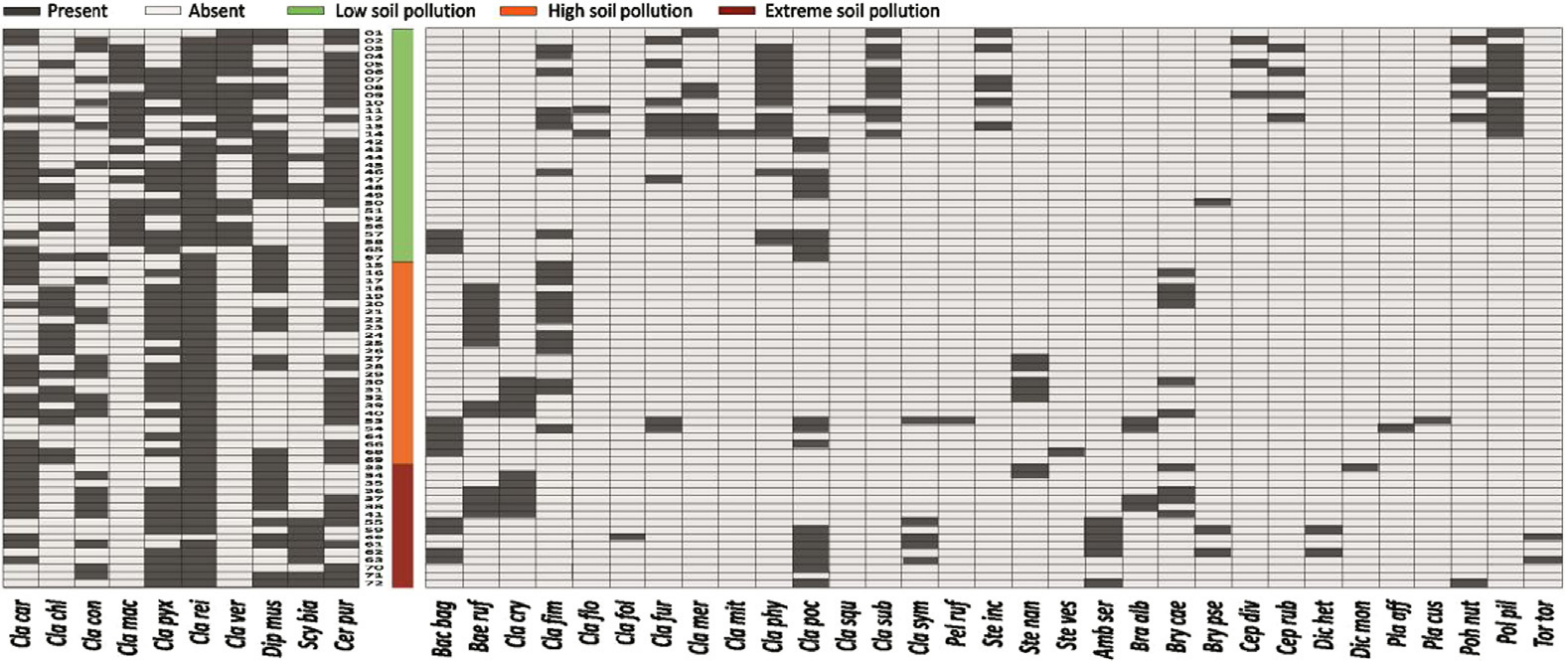
Species presence matrix in the studied plots; the plots are arranged according to soil pollution classes. Dominants, species recorded in no less than half of the plots, and simultaneously with mean cover higher than 2% within at least one of the pollution classes, are separated on the left side. For abbreviations of species see Table 3.

**Fig. 3:**
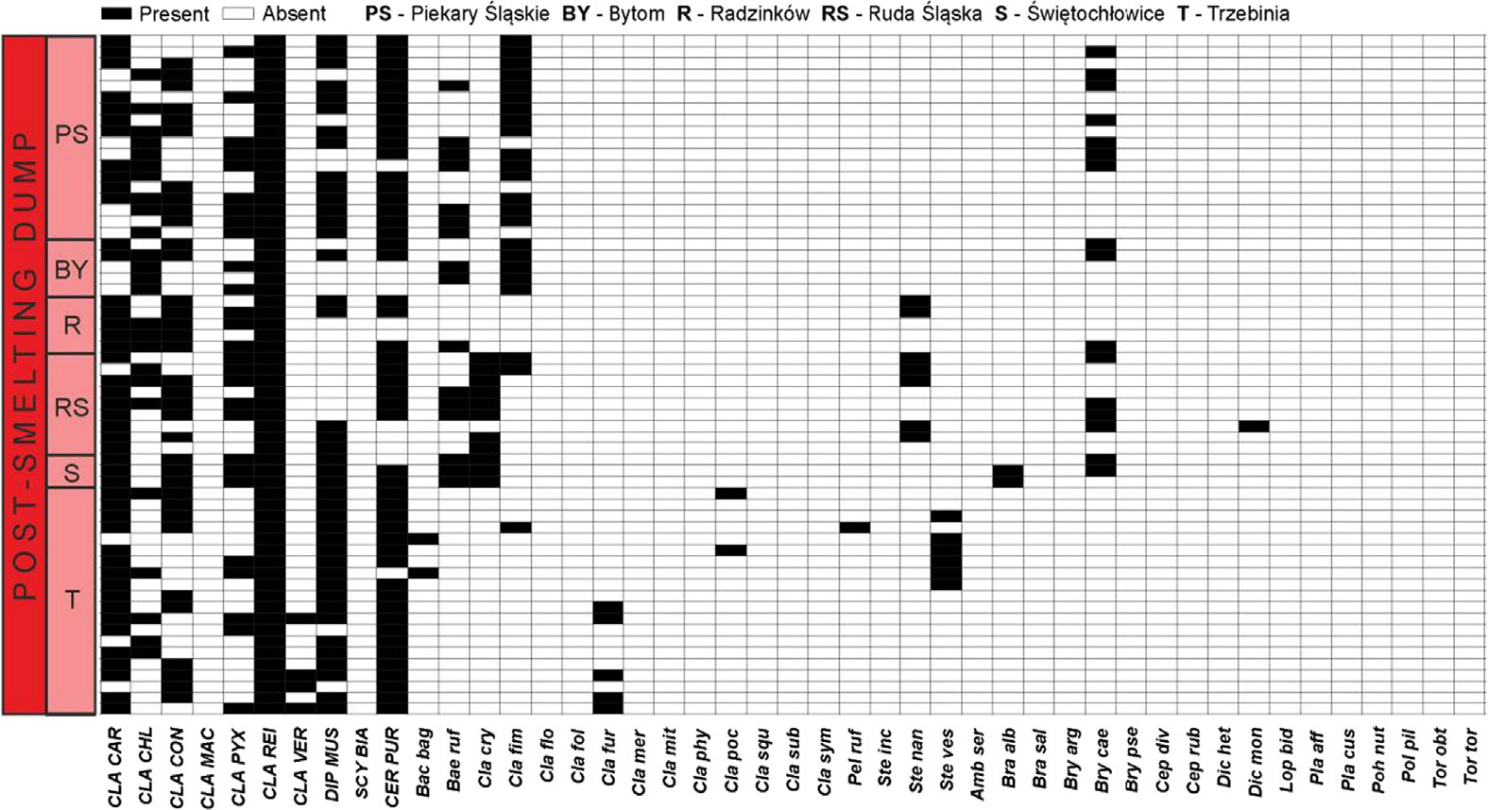
Species presence matrix in the plots representing post-smelting dumps. Dominant species are marked in capital letters. For abbreviations of species see Table 3; for abbreviations of study sites see Fig. 1.

**Fig. 4:**
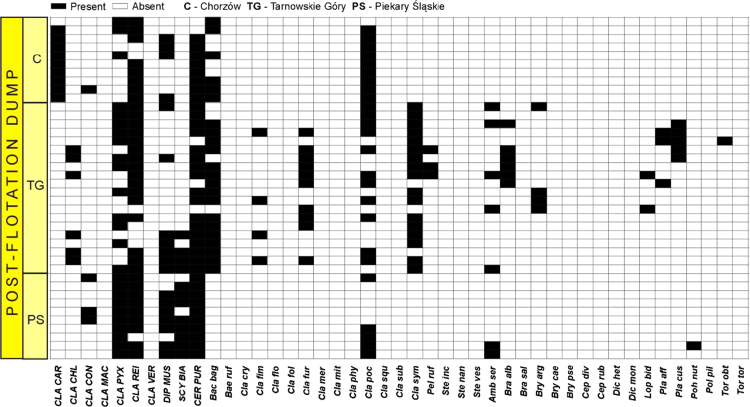
Species presence matrix in the plots representing post-flotation dumps. Dominant species are marked in capital letters. For abbreviations of species see Table 3; for abbreviations of study sites see Fig. 1.

**Fig. 5:**
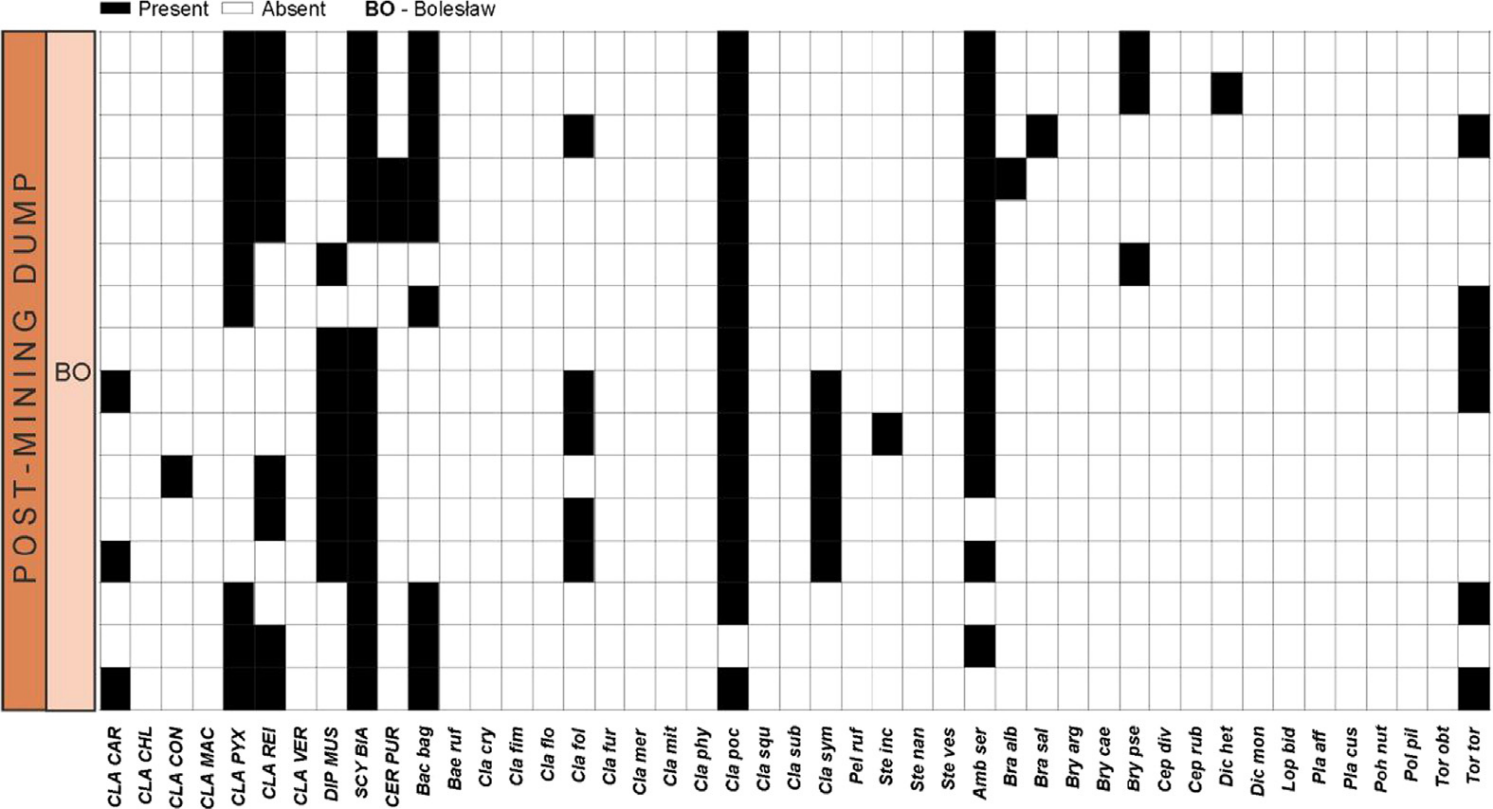
Species presence matrix in the plots representing post-mining dumps. Dominant species are marked capital letters. For abbreviations of species see Table 3; for abbreviations of study sites see Fig. 1.

**Fig. 6:**
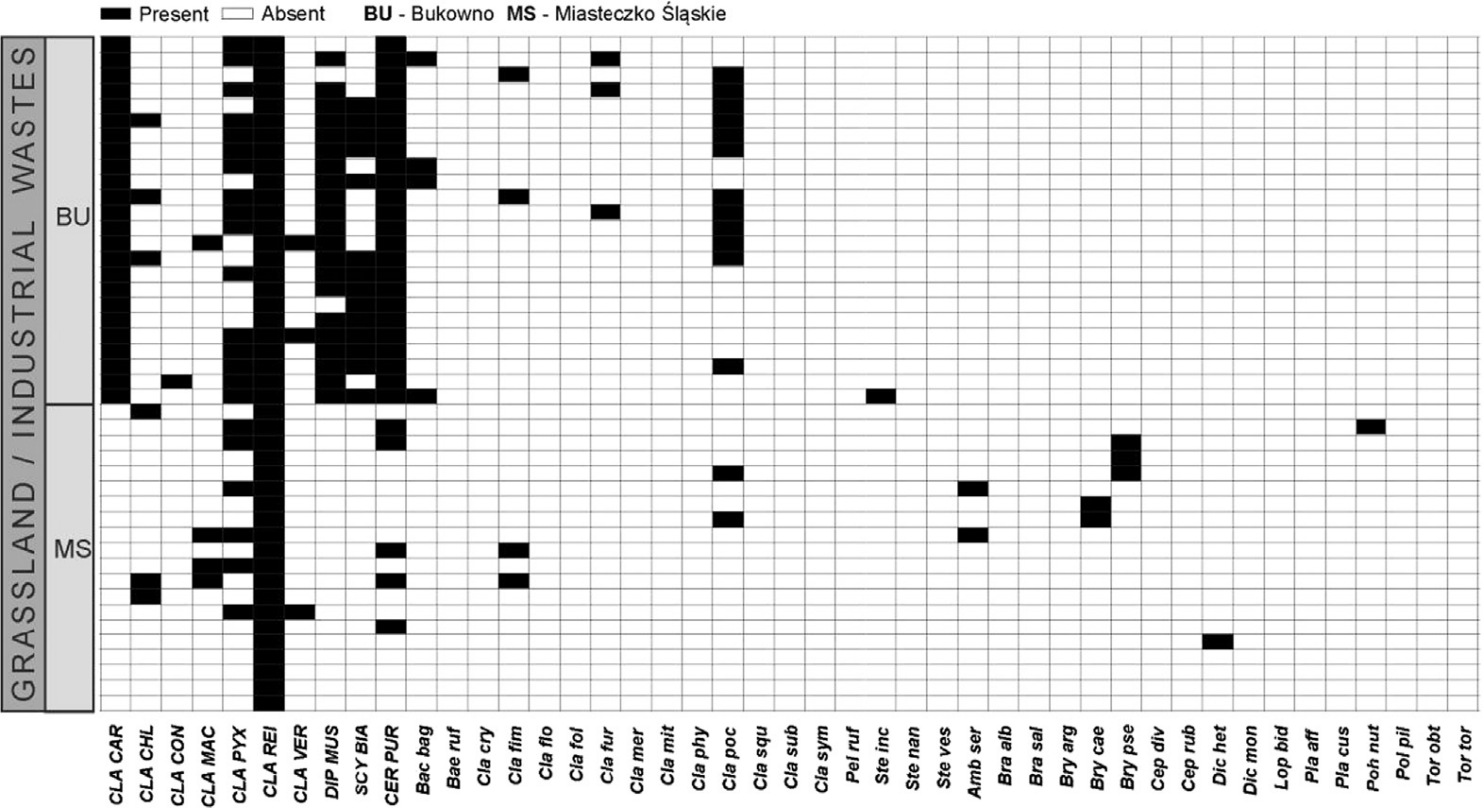
Species presence matrix in the plots representing grassland/industrial wastes – smelter environ habitat type. Dominant species are marked capital letters. For abbreviations of species see Table 3; for abbreviations of study sites see Fig. 1.

**Fig. 7:**
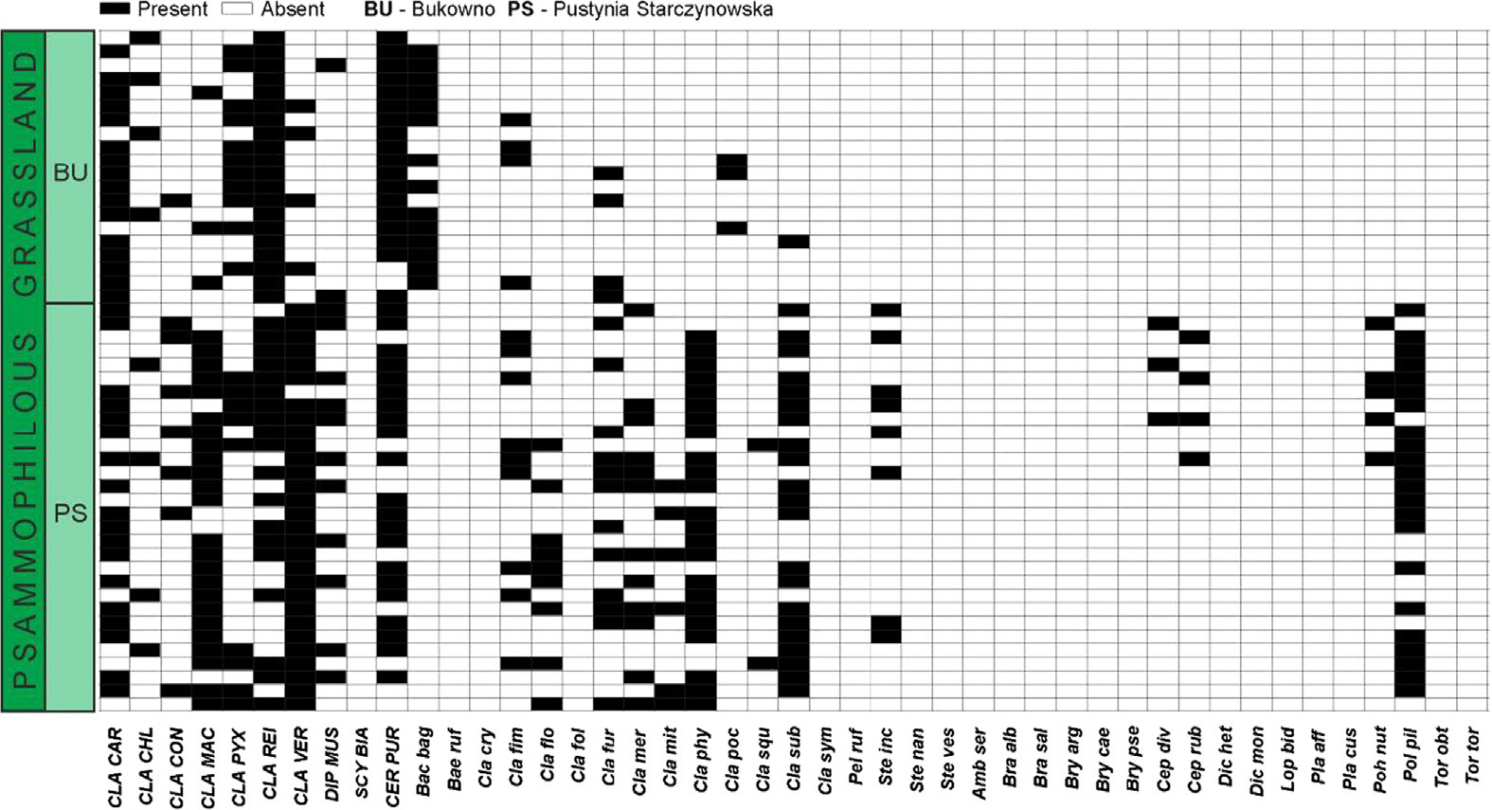
Species presence matrix in the plots representing psammophilous grasslands. Dominant species are marked in capital letters. For abbreviations of species see Table 3; for abbreviations of study sites see Fig. 1.

Data on the specific structure of cryptogamic communities in relation to soil chemical parameters in sites directly associated with the processing of Zn–Pb ores in southern Poland are presented (Fig. 1). Different types of anthropogenic and semi-natural habitats, i.e. post-smelting, post-flotation, post-mining dumps, grassland or industrial wastes in smelter environs and psammophilous grassland, were considered. Analysis of cryptogamic biota within study plots with respect to the chemical parameters of the corresponding soil resulted in identification of three different pollution classes related to the concentration of heavy metals: low, high, and extreme (for details see Ref. [1]). The ranges of analysed chemical parameters for each class are presented in Table 1 and for particular habitat types in Table 2. As regard cryptogamic biota, altogether, 45 species, including 27 lichens and 18 bryophytes, were recorded (Table 3). The presence of particular species in plots assigned to certain soil pollution class are shown in Fig. 2; whereas the presence in study plots representing particular habitat types in Fig. 3, Fig. 4, Fig. 5, Fig. 6, Fig. 7. Details related to the determination of soil pollution classes and their chemical and biotic characteristics can be found in Ref. [1].

**Table 1:** Descriptive statistics for soil chemical parameters, species richness and coverage of lichens and bryophytes for particular soil pollution classes.

Soil pollution class	Low		High		Extreme	
	Mean±SD	Min–Max	Mean±SD	Min–Max	Mean±SD	Min–Max
Zn (mg kg^−1^)	527.0±580.9	92.4–2747.0	31727.9±12342.0	11383.4–54581.3	79824.1±14653.9	60296.1–100792.4
Pb (mg kg^−1^)	195.0±201.1	50.4–792.2	13837.1±7057.7	2337.8–24880.0	14299.4±8257.3	2157.6–23192.5
Cd (mg kg^−1^)	6.0±4.5	2.0–17.8	149.6±117.8	6.2–366.3	213.6±158.2	21.0–520.5
As (mg kg^−1^)	18.4±45.5	2.2–232.3	3024.1±3832.7	53.6–14815.5	2352.0±1573.0	100.4–4665.5
Zn-ex (mg kg^−1^)	173.8±191.5	6.2–669.3	1491.8±1905.8	38.6–5723.5	5676.0±4096.9	785.3–11076.2
Pb-ex (mg kg^−1^)	120.4±149.4	32.2–772.0	1300.3±1869.0	15.3–8099.6	2867.1±2605.8	452.8–7328.6
Cd-ex (mg kg^−1^)	3.5±4.2	0.2–15.7	22.3±20.4	0.9–73.0	74.1±67.2	1.9–183.6
As-ex (mg kg^−1^)	0.5±1.8	0.0–9.8	15.0±63.6	0.1–326.1	0.6±0.6	0.1–1.9
C_org._ (%)	1.6±1.5	0.2–5.9	4.2±2.6	1.0–10.0	4.0±2.4	1.3–8.4
N_tot._ (%)	0.1±0.1	0.0–0.4	0.2±0.2	0.1–0.9	0.1±0.1	0.0–0.3
C/N	19.4±11.2	6.3–52.5	22.7±6.2	8.8–33.5	62.5±72.6	10.9–271.3
pH	5.3±1.1	4.0–7.1	7.0±0.5	6.2–7.9	6.7±0.4	6.3–7.3
Number of lichen species	6.6±1.9	2.0–10.0	5.2±1.2	2.0–7.0	5.6±1.1	4.0–7.0
Number of bryophyte species	1.6±1.1	0.0–4.0	1.1±0.8	0.0–3.0	1.8±1.0	0.0–3.0
Lichen coverage (%)	52.5±20.8	19.8–85.4	66.4±33.7	11.5–90.5	38.4±16.3	14.6–68.0
Bryophyte coverage (%)	13.2±14.8	0.0–62.5	12.5±11.7	0.0–37.5	22.3 ±17.2	0.0–62.5

**Table 2:** Descriptive statistics for soil chemical parameters, species richness and coverage of lichens and bryophytes for particular habitat types.

Habitat type	Post-smelting dumps		Post-flotation dump		Post-mining dump		Grassland/industrial wastes		Psammophilous grassland	
	Mean±SD	Min–Max	Mean±SD	Min–Max	Mean±SD	Min–Max	Mean±SD	Min–Max	Mean±SD	Min–Max
Zn (mg kg^−^^1^)	44844.9±25200.7	2097.1–99720.6	46698.6±40691.4	2747.0–100792.4	68263.6±7782.7	60296.1–79096.0	637.1±221.6	161.5–928.9	232.9±136.4	92.4–531.9
Pb (mg kg^−^^1^)	15866.72±7035.5	641.0–24880.0	11019.5±7006.9	755.6–19113.8	2954.7±576.2	2157.6–3752.5	203.1±198.1	105.2–792.2	130.5±110.5	50.4–503.7
Cd (mg kg^−^^1^)	127.5±117.9	5.3–366.3	258.7±180.8	16.6–520.5	235.4±28.2	197.6–263.8	8.7±4.7	4.7–17.8	3.7±2.2	2.0–9.1
As (mg kg^−^^1^)	3395.1±3507.7	103.5–14815.5	1198.1±1216.7	53.6–2850.9	794.4±199.9	596.7–1036.8	9.1±6.9	2.2–26.6	6.0±3.8	2.8–19.5
Zn-ex (mg kg^−^^1^)	1272.1±1734.8	38.6–5723.5	5015.5±3663.4	669.3–11076.2	9410.9±992.3	8272.3–10900.7	270.0±153.3	117.6–641.0	87.5±147.3	6.2–496.3
Pb-ex (mg kg^−^^1^)	1165.4±1461.8	15.3–6013.2	4580.1±2952.2	436.7–8099.6	832.5±232.7	452.8–1066.4	150.5±207.6	49.5–772.0	76.2±61.9	32.2–267.2
Cd-ex (mg kg^−^^1^)	15.4±14.7	0.9–61.8	99.3±71.2	11.3–183.6	85.0±8.7	72.6–95.8	5.9±4.8	1.9–15.7	1.5±2.3	0.2–7.3
As-ex (mg kg^−^^1^)	13.3±59.3	0.1–326.1	0.7±0.6	0.1–1.9	1.0±0.5	0.4–1.6	0.3±0.3	0.1–0.9	0.1±0.02	0.02–0.1
C_org._ (%)	4.4±2.7	1.0–9.9	3.5±1.9	1.3–7.6	2.5±0.8	1.6–3.6	1.3±0.9	0.4–3.8	1.7±1.9	0.2–5.9
N_tot._ (%)	0.2±0.1	0.1–0.3	0.2±0.3	0.01–0.9	0.2±0.1	0.1–0.2	0.1±0.05	0.04–0.2	0.1±0.1	0.01–0.4
C/N	28.9±11.6	11.5–52.9	76.5±96.1	8.8–271.3	14.4±3.8	10.9–20.0	14.0±5.4	6.3–24.6	22.9±13.2	6.7–52.5
pH	6.8±0.6	6.2–7.9	7.0±0.3	6.5–7.3	7.0±0.1	6.9–7.1	6.2±0.9	4.4–7.1	4.6±0.5	4.0–5.5
Number of lichen species	5.5±1.1	4.0–7.0	4.8±1.4	2.0–7.0	5.8±0.8	5.0–7.0	5.3±1.6	2.0–7.0	7.6±1.7	4.0–10.0
Number of bryophyte species	1.1±0.8	0.0–3.0	1.6±1.0	0.0–3.0	2.2±0.8	1.0–3.0	0.9±0.5	0.0–2.0	2.2±1.1	1.0–4.0
Lichen coverage (%)	63.1±32.6	14.6–89.3	37.1±19.6	11.5–71.3	34.2±12.5	27.3–56.3	62.23±25.58	19.8–85.4	49.0±14.9	24.85–77.70
Bryophyte coverage (%)	16.2±16.9	0.0–62.5	14.4±12.0	0.0–38.0	29.8±11.8	17.5–46.3	5.85±4.58	0.0–11.3	14.7±13.9	0.30–46.25

**Table 3:** List of recorded lichen and bryophyte species and their general characteristics.

Species	Species abbreviation	Functional group^a^	Presence in particular soil pollution classes^b^	Presence in particular habitat types^c^	General frequency in all studied plots (%)	Mean cover in all studied plots (%)
LICHENS						
*Bacidia bagliettoana*	*Bac bag*	crust/ap	●●●	●●●●●	24.29	0.812
*Baeomyces rufus*	*Bae ruf*	crust/ap	○●●	○○●○○	7.62	0.069
*Cladonia cariosa*	*Cla car*	dimor/ap	●●●	●●●●●	55.24	4.648
*Cladonia cervicornis* subsp. *verticillata*	*Cla ver*	dimor/ap	●○○	●●●○○	19.05	1.421
*Cladonia chlorophaea*	*Cla chl*	dimor/ap	●●○	●●●●○	21.90	0.588
*Cladonia conista*	*Cla con*	dimor/so	●●●	●●●●○	22.38	0.719
*Cladonia cryptochlorophaea*	*Cla cry*	dimor/ap	○●●	○○●○○	5.24	0.033
*Cladonia fimbriata*	*Cla fim*	dimor/so/ap	●●○	●●●●●	21.43	0.376
*Cladonia floerkeana*	*Cla flo*	dimor/ap	●○○	●○○○○	4.29	0.060
*Cladonia foliacea*	*Cla fol*	squam/ve	○○●	○○○○●	2.38	0.098
*Cladonia furcata*	*Cla fur*	dimor/ap/ve	●●○	●●●●●	17.14	1.340
*Cladonia macilenta*	*Cla mac*	dimor/ap	●○○	●●○○○	14.76	0.743
*Cladonia merochlorophaea*	*Cla mer*	dimor/so	●○○	●○○○○	5.71	0.098
*Cladonia mitis*	*Cla mit*	frut/ve	●○○	●○○○○	2.86	0.110
*Cladonia phyllophora*	*Cla phy*	dimor/ve/ap	●○○	●○○○○	10.95	0.729
*Cladonia pocillum*	*Cla poc*	dimor/ap	●●●	●●●●●	28.10	1.257
*Cladonia pyxidata*	*Cla pyx*	dimor/ap	●●●	●●●●●	49.52	3.562
*Cladonia rei*	*Cla rei*	dimor/so	●●●	●●●●●	88.10	23.381
*Cladonia squamosa*	*Cla squ*	dimor/so	●○○	●○○○○	0.95	0.029
*Cladonia subulata*	*Cla sub*	dimor/so	●○○	●○○○○	10.48	0.748
*Cladonia symphycarpa*	*Cla sym*	squam/ap	○●●	○○○●●	10.48	0.569
*Diploschistes muscorum*	*Dip mus*	crust/ap	●●●	●●●●○	47.62	2.483
*Scytinium biatorinum*	*Scy bia*	crust/ap	●○●	○●○●●	19.05	1.076
*Peltigera rufescens*	*Pel ruf*	fol/ap	○●○	○○●●○	1.90	0.083
*Stereocaulon incrustatum*	*Ste inc*	frut/ap	●○○	●●○○●	4.76	0.171
*Stereocaulon nanodes*	*Ste nan*	frut/ap	○●●	○○●○○	3.33	0.019
*Stereocaulon vesuvianum*	*Ste ves*	frut/ap	○●○	○○●○○	2.86	0.081

BRYOPHYTES						
*Amblystegium serpens*	*Amb ser*	RM/PS	○○●	○●○●●	10.48	0.940
*Brachythecium albicans*	*Bra alb*	RM/PS	○●●	○○●●●	4.29	0.426
*Brachythecium salebrosum*	*Bra sal*	RM/C	○○○	○○○○●	0.48	0.010
*Bryum argenteum*	*Bry arg*	ST/C	○○○	○○○●○	1.90	0.033
*Bryum caespiticium*	*Bry cae*	ST/C	○●●	○●●○○	8.57	0.914
*Bryum pseudotriquetrum*	*Bry pse*	ST/PS	●○●	○●○○●	2.86	0.390
*Cephaloziella divaricata*	*Cep div*	ST/C	●○○	●○○○○	1.43	0.007
*Cephaloziella rubella*	*Cep rub*	ST/C	●○○	●○○○○	1.90	0.014
*Ceratodon purpureus*	*Cer pur*	ST/C	●●●	●●●●●	73.33	9.302
*Dicranella heteromalla*	*Dic het*	ST/C	○○●	○●○○●	0.95	0.019
*Dicranum montanum*	*Dic mon*	ST/PS	○○●	○○●○○	0.48	0.083
*Lophocolea bidentata*	*Lop bid*	PS	○○○	○○○●○	0.95	0.019
*Plagiomnium affine*	*Pla aff*	RM/PS	○●○	○○○●○	1.43	0.048
*Plagiomnium cuspidatum*	*Pla cus*	RM/PS	○●○	○○○●○	2.38	0.048
*Pohlia nutans*	*Poh nut*	ST/C	●○●	●●○●○	3.33	0.043
*Polytrichum piliferum*	*Pol pil*	TT/PS	●○○	●○○○○	10.48	0.907
*Tortella tortuosa*	*Tor tor*	ST/PS	○○●	○○○○●	2.86	0.717
*Tortula obtusifolia*	*Tor obt*	C	○○○	○○○●○	0.48	0.167

• – present, ○ – absent

a For lichens – growth forms (specified on the basis of the most frequently observed form): crust – crustose; fol – foliose; squam – squamulose; frut – fruticose; dimor – dimorphic (squamulose primary thallus and fruticose secondary thallus); main reproduction type according to Ref. [2]: ap – sexual reproduction by apothecia; so – vegetative reproduction by soredia and isidia; ve – vegetative reproduction by thallus fragmentation. For bryophytes: growth forms according to Ref. [3]: RM, rough mat; ST, short turf; TT, tall turf; life history strategy according to Ref. [3]: C, colonist; PS, perennial stayer.

b Low, high and extreme; respectively.

c ‘Psammophilous grassland’, ‘Grassland/industrial wastes - smelter environs’, ‘Post-smelting dumps’, ‘Post-flotation dump’, ‘Post-mining dump’, respectively.

## 2 Experimental design, materials, and methods

### 2.1 Field studies and sampling

The fieldwork was conducted in the Silesia-Cracow Upland area, one of the most polluted regions in Poland, associated for centuries with the processing of Zn–Pb ores (Fig. 1). The sampling were conducted in the summer seasons of 2015 and 2016. Altogether, 210 plots, 1 m×1 m, representing homogenous patches of vegetation, were examined with respect to the presence and coverage of lichen and bryophyte species. The size of the plots is considered appropriate for a detailed survey of cryptogamic biota (see Refs. [4], [5]). The following cover-abundance scale was used ([6], modified): r, <1% or 1–2 individuals; +, <5% cover or 3–5 individuals;1a, <5% cover and several individuals; 1b, <5% cover and frequent; 2a, cover 5–12.5%; 2b, cover 12.5–25%; 3, cover 25–50%; 4, cover 50–75% and 5, cover 75–100%. The species were identified in the field only in cases of specimens whose taxonomic classification was not problematic. Most individuals, however, were collected for precise determination based on a detailed examination of their morphology and, in the case of lichens, chemical features. Lichen secondary substances, required for the identification of certain species, were determined by means of TLC, following [7]. The nomenclature follows [8] and [9] for lichens and bryophytes, respectively. Additionally, percentage of total coverage of lichens and bryophytes was estimated for each plot. From 72 plots three soil subsamples, to a depth of 5 cm, were collected and bulked in one composite sample.

### 2.2 Chemical analysis of soil samples

The soil samples were dried and passed through a 2-mm sieve. Acidity (pH) was electrometrically determined in 1-M KCl suspensions with a Hach Lange HQ40d pH meter. Organic carbon content was measured using the dry combustion technique with a LECO SC-144DR Analyzer (LECO Corp., MI, USA) and total N content using the Kjeldahl method using Kjeltec 2300 Analyzer Unit (FOSS Tecator, Sweden). Soil samples (5 g DW) were digested with 70% HClO_4_ (Merck, Suprapur) using a digester (FOSS Tecator 2020, Sweden). Subsequently, flame atomic absorption spectrometry using Varian Fast Sequential Atomic Absorption Spectrometer 280 (Varian, Australia) for Zn, Cd, Pb and Varian Zeeman Atomic Absorption Spectrometer 280 with Graphite Tube Atomizer 120 (Varian, Australia) for As was applied. Exchangeable forms of elements were determined by extracting 5 g DW with a 0.05-M EDTA solution and measured by means of flame atomic absorption spectrometry. Certified reference materials (CRM048–50G Sigma-Aldrich, BCR-483 Sigma-Aldrich, ISE-912 WEPAL – Wageningen University) were used for quality assurance. Appropriate solutions without samples were used as reagent blanks. The analyses were repeated three times and the mean values considered as one observation.
