# Supplementary material for: Data on cryptogamic biota in relation to heavy metal concentrations in soil
Source: Data Brief. 2018 May 31;19:1110–9. doi: 10.1016/j.dib.2018.05.137 (PMC6140828; doi:10.1016/j.dib.2018.05.137)
Supplement: Supplementary file 1 — Supplementary material [file mmc1.doc]

Manuscript Number: DIB-D-18-00825

Declarations of interest: none
